# Treatment of Dichlorethyl-Sulphide (“Mustard Gas”) Injuries

**Published:** 1919

**Authors:** 


***Treatment of Dichlorethyl-Sulphide ("Mustard Gas") Injuries.*** By Aldred S. Warthin, and associates. *Journal of Laboratory and Clinical Medicine*, October 1918.

Authors review briefly literature on treatment of mustard gas lesions. The authors recommend the following treatment from their own practice: —

*a*) *Mild Injuries.* Wash immediately or, better, immerse parts one-half to two hours in Dakin's solution (strength of about 0.5 per cent. hypochlorous acid). If too irritating (not likely if skin is unbroken), dilute solution or shorten time of immersion. For genital lesions, use sitz bath; for extensive lesions, full bath. When immersion of parts is not practicable, use wet Dakin's dressings or irrigate with Dakin's solution. If wet treatment cannot be carried out, application of dichloramin-T in chlorcosane, or chloramine T in sodium stearate may be substituted. After Dakin's solution, use wet dressings, irrigation or bath of sterile hypertonic saline

(5 to 10 per cent.), alternating with the above Dakin's solution and a sterile physiologic solution, according to judgment. Continue this treatment during the day as long as lesions remain erythematous. At night dry skin aseptically, apply a vanishing surgical cream containing hypochlorous acid or liberating chlorine of the type of chlorazene surgical cream, or use wet hypertonic saline dressings. Avoid use of dusting powders.

In case of vesicle formation, empty vesicles early under aseptic precautions with hypodermic syringe or sterile needle, and allow intact vesicle wall to collapse.

In case of denuded surfaces with much pain, and when there are bedsores, the colloidal saline bath is indicated. To prepare bath, dissolve one pound of commercial cornstarch and one pound of sodium bicarbonate in 20 to 30 gallons of sterile physiologic salt solution, at a temperature of 90° to 95°. Patient can be left immersed in this from 15 minutes to 48 hours, but must be carefully watched to avoid heart weakness.

*Mild Conjunctivitis.* Saturated boric acid or hot vapor baths; also use of 0.5 per cent. solution dichloramine-T in chlorcosane.

*Mild Respiratory Lesions.* For mouth and throat a weak Dakin's or a .25 to .50 per cent. chlorazene solution. For aphonia use ice bag externally and steam inhalations with or without compound tincture of benzoin.

*b*) *Severe Injuries.* For skin lesions with necrosis, follow treatment outlined above, full baths, irrigation, slush or sponge bath, or wet packs of Dakin's solution alternating with physiologic and hypertonic salines, or employ colloidal bicarbonate bath constantly. With lesions of shoulders, buttocks etc., patient should be hung in canvas body cradle for constant colloidal or saline bath. Where decubitus or sloughs occur, remove necrotic tissue with sterile forceps or frequent hot irrigations, using saline, Dakin's 1 : 10,000 potassium permanganate solutions, etc. Hyperchlorous solution made according to Carrel's method may be used with good results. When patients are weak and infection progressing, tepid sponge baths of above solution may be used.

*Shocks* should be treated as surgical shock.

*Kidney complications* in severe cases may be treated by Murphy drip. Low protein salt-free diet. Sodium carbonate may be given internally. Digipuratum of value. Keep up intake of water.

*Heart complications* treated by active digitalis compounds.

Digipuratum 1.5 grams 3 to 4 times daily benefited many cases.

*Lungs.* No specific treatment for primary lesions. For secondary infections, treat according to infecting organisms, usually the streptococcus.

*Gastro-intestinal.* Liquid low protein diet; sodium carbonate is indicated.

*Furunculosis.* Open and drain large furuncles and give a stock, if possible, of autogenous staphylococcus vaccine in large doses, 1 billion every third day for four injections.

*Eye Injuries.* See previous article on "Ocular Lesions Produced by Mustard Gas", by same authors.

